# Analysis of the autoimmune response to BP180 in Chinese stroke patients^☆^

**DOI:** 10.1016/j.abd.2022.01.012

**Published:** 2022-11-28

**Authors:** Jing Wang, Hong Liu, Zhenzhen Wang, Qing Pan, Furen Zhang

**Affiliations:** a Shandong Provincial Hospital for Skin Diseases, Cheeloo College of Medicine, Shandong University, Jinan, Shandong, China; b Department of Dermatology and Venereology, the Second Affiliated Hospital, Anhui Medical University, Hefei, Anhui, China

**Keywords:** Antibodies, Bullous pemphigoid, Stroke

## Abstract

**Background:**

Significant association between bullous pemphigoid (BP) and stroke has been reported. This study aimed to evaluate the level of anti-BP180 antibody in stroke patients to explore the relationship between BP and stroke in their pathogenesis.

**Methods:**

We collected serum samples from stroke patients and matched controls between February 2019 and June 2020. The anti-BP180 antibody levels were measured by enzyme-linked immunosorbent assay (ELISA).

**Results:**

A total of 1183 stroke patients including 970 with cerebral infarction (CI), 192 with intracerebral hemorrhage (ICH), 21 with CI and ICH, and 855 controls were enrolled in this study. Anti-BP180 autoantibody values were significantly higher in stroke patients than in controls (p < 0.001). Anti-BP180 autoantibody-positive rates were 12.51% (148) in stroke patients and 4.68% (40) in controls (p < 0.001, OR = 2.65). In anti-BP180 autoantibody-positive subjects, the values were significantly higher in stroke patients than in controls (p < 0.001). However, only 10 (6.76%) stroke patients and 3 (7.5%) controls had high values (> 100 RU/mL) (p = 0.87). Stratified analysis showed that anti-BP180 antibody positive rates were independent of age, sex, and stroke subtypes in the stroke group. Positive rates in patients with both CI and ICH were nearly two times higher than those in patients with either CI or ICH alone (p = 0.11, OR = 1.94).

**Study limitations:**

This study had a limited sample size and lacked quantitative criteria for stroke severity.

**Conclusions:**

Anti-BP180 antibody values and positive rates were higher in stroke patients than in controls, suggesting that stroke patients may have higher of developing BP.

## Introduction

Bullous Pemphigoid (BP) is a chronic autoimmune bullous disease characterized by subepidermal blistering, mainly affecting older people. BP incidence increases with age, and 190‒312 cases per million people older than 80 years are observed every year.^1^ BP180 and BP230 are two major antigens of BP. The NC16A domain of BP180 plays vital roles in BP pathogenesis due to its direct binding to the autoantibody.^2^

BP180 is expressed in the skin and nervous system.^3^ The association between BP and Neurological Disease (ND) has been reported, especially stroke. Cordel et al. found that 35% of BP patients had concomitant ND, of which 55% presented with dementia and 42% presented with stroke.^4^ The study conducted by the Dermatology Department of The Central European University indicated that the ND prevalence rate in BP was 27.66%, and stroke was most common.^5^ Exposure of antigenic nerve epitope and cross-reactivity of autoantibodies in ND may explain the relationship between BP and stroke.^6^

However, to date, only one study with a small sample size has found anti-BP180 antibodies in 14% (14/100) of stroke patients and 5% (5/100) of controls using ELISA (p < 0.05).^7^ Therefore, to further explore the correlations between BP and stroke, it is important to investigate the prevalence of anti-BP180 antibodies in patients with stroke. Herein, we analyzed the prevalence of anti-BP180 antibodies in 1183 stroke patients and 855 controls in a Chinese Han population.

## Materials and methods

The Ethics Committee of hospital approved this study. All subjects provided written informed consent. All samples were collected from one hospital of Medical University between February 2019 and June 2020. Venous blood (2 mL) was collected, and the serum was separated.

A commercially available ELISA kit (EUROIMMUN, Germany) was used to detect anti-BP180 IgG autoantibodies in the sera of patients and healthy controls (human BP180 NC16A domain). The evaluation was based on the cutoff value of 20 RU/mL: ≥ 20 was positive; < 20 was negative. The data were analyzed by SPSS 22 software. The qualitative data, such as sex (male/female), and BP180 antibody (positive/negative) were statistically analyzed by the Chi-Square test. The quantitative data, such as age, which had Gaussian distribution were statistically analyzed by a two-sided unpaired Student’s *t*-test. The quantitative data, such as BP180 antibody values, which did not conform to Gaussian distribution were statistically analyzed by a two-sided Mann-Whitney *U* test.

## Results

Among the 1183 stroke patients (717 males, 466 females, average age 64.85 ± 13.08 years), 970 had Cerebral Infarction (CI), 192 had Intracerebral Hemorrhage (ICH), and 21 had both CI and ICH. The 855 healthy controls included 523 males and 332 females, with an average age of 63.99 ± 13.03 years. There was no significant difference in age and sex distribution between the stroke and control groups (Table 1).

**Table 1:** Comparison of sex, age and anti-BP180 antibody values between the stroke patients and controls.

Item	Stroke	Control	p-value	OR
n	1183	855		
Sex, M/F	717/466	523/332	0.80	
Age	64.85 ± 13.08	63.99 ± 13.03	0.14	
BP180 (RU/mL)	0 (0‒209.90)	0 (0‒191.33)	<0.001	
BP180 (≥20 RU/mL)	37.55 (20.56‒209.90)	32.18 (20.25‒191.33)	<0.001	
BP180 +/− (%)	148/1035 (12.51/87.49%)	40/815 (4.68/95.32%)	<0.001	2.65
20‒100/>100 RU/mL (%)	138/10 (93.24/6.76%)	37/3 (92.5/7.5%)	0.87	1.01

Age is given as mean and standard deviation. Values are given as median (range). BP180 +/− represented BP180 positive (≥20 RU/mL)/BP180 negative (<20 RU/mL). The p-values were calculated by comparing the data between the two groups. A p > 0.05 indicated no significance. OR, Odds Ratio.

**Figure 1:**
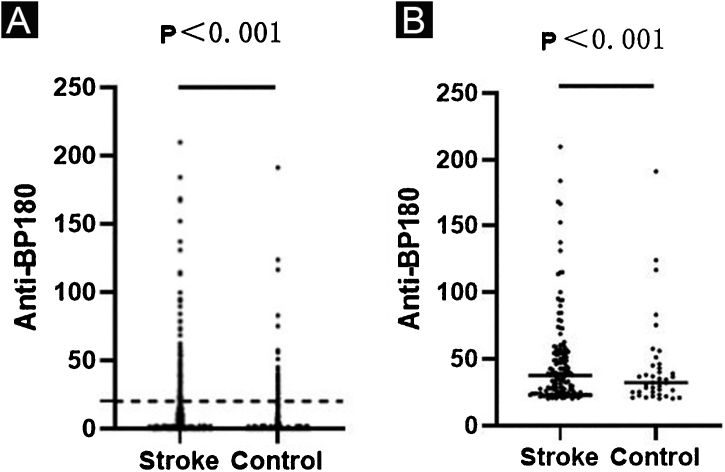
ELISA results in the stroke and control groups. Dots indicate the index value of each sample. (A) Anti-BP180 antibody values of 1183 stroke patients and 855 controls. The dotted lines represent the cutoff value of 20 RU/mL. (B) Anti-BP180 antibody-positive values of 148 stroke patients and 40 controls. Black lines represent the median.

Anti-BP180 autoantibody values were significantly higher in the stroke group than in the control group (p < 0.001) (Table 1, Fig. 1a). Anti-BP180 autoantibody-positive rate was 12.51% (148) in stroke patients and 4.68% (40) in controls, with significant difference (p < 0.001, OR = 2.65) (Table 1). In anti-BP180 autoantibody-positive subjects, the values were also higher in the stroke group (37.55 RU/mL, range 20.56‒209.90 RU/mL) compared with the control group (32.18 RU/mL, range 20.25‒191.33 RU/mL) (p < 0.001) (Table 1, Fig. 1b). Notably, only 10 (6.76%) stroke patients and 3 (7.5%) controls showed high values (> 100 RU/mL), but without significant difference between them (p = 0.87, OR = 1.01) (Table 1).

Stratified analysis of anti-BP180 antibody positive rate in the stroke group showed no significant difference in age, sex, and stroke subtypes (p > 0.05). There had a similar positive rate between the three-stroke subtypes (p = 0.11) (Table 2).

**Table 2:** ELISA results of the anti-BP180 antibody positive rate in the stroke group.

Stroke	BP180+/- (%)	p-value	X^2^	OR	95% CI
Age					
≤60 years	49/364 (11.86/88.14%)				
>60 years	99/671 (12.86/87.14%)	0.62	0.24	0.92	0.67‒1.28
Gender					
Male	85/632 (11.85/88.15%)				
Female	63/403 (13.52/86.48)	0.40	0.72	0.88	0.65‒1.19
Type					
CI	121/849 (12.47/87.53%)				
ICH	22/170 (11.46/88.54%)				
CI and ICH	5/16 (23.81/76.19%)	0.27	2.65		
Type					
CI and ICH	5/16 (23.81/76.19%)				
CI or ICH	143/1019 (12.31/87.69%)	0.11	2.50	1.94	0.89‒4.22

The numbers and percentages of anti-BP180 antibody by ELISA are based on the cutoff value of ≥20 RU/mL. The p-value, X^2^, OR and 95% CI are indicated for each comparison. A p > 0.05 was considered to indicate no statistical significance.

CI, Cerebral Infarction; ICH, Intracerebral Hemorrhage; OR, Odds Ratio; 95% CI, 95% Confidence Interval.

## Discussion

This study on a relatively large sample of stroke patients suggested that monitoring of anti-BP180 antibody is critical for prospective BP prevention and provides novel insights into the relationship between stroke and BP.

BP and stroke are interrelated risk factors, but their sequential relationship remains controversial. One hypothesis is that ND precedes BP in months or years.^8^ Nerve damage exposes the central nervous system BP180 to the immune system, and this exposure may lead to autoantibodies that cross-react with the skin’s basal membrane area leading to BP.^6^ Another hypothesis is that stroke is different from general ND and belongs to cerebrovascular diseases and often occurs after BP. Given the BP-derived inflammation and increased eosinophilia in blood and tissues, the high coagulation state of anti-phospholipid antibodies worsens existing atherosclerosis, promotes thrombosis, and causes a stroke.^9^

This study demonstrated that anti-BP180 autoantibody values and positive rate were higher in stroke patients than in controls. The risk of anti-BP180 antibody prevalence was 2.65 times higher in the stroke group than in the control group. However, for those with positive anti-BP180 antibody in the two groups, BP did not occur in the 6-month follow-up to date, which is consistent with the findings of Kokkonen et al.,^10^ who reported anti-BP180 antibody in 18% of Alzheimer's patients and 3% of controls, but no BP-like skin lesions. There are several possible reasons for this finding. First, the values of anti-BP180 antibody in stroke patients were too low to activate the immune response of the complement system and other cytokines, and could not induce BP. In the present study, most of the anti-BP180 antibody-positive subjects had low values. Second, some patients may be missed due to atypical lesions, such as pruritus or erythema, without blisters. Third, the follow-up time was not sufficiently long to develop BP. Therefore, whether anti-BP180 antibody-positive subjects could develop BP requires longer follow-up.

Younger stroke patients are significantly more likely to develop BP180 serum autoreactivity than older stroke patients.^7^ However, in this study, the anti-BP180 antibody positive rate was not significantly different between stroke patients aged ≤ 60 and > 60 years. Another study on anti-BP180 antibodies in non-BP populations found that antibody incidence does not change significantly depending on age or sex.^11^ Therefore, stroke has a more significant impact on anti-BP180 antibodies than age and sex.

There was no statistically significant difference in the anti-BP180 antibody positive rate between the three-stroke subtypes. However, it should be noted that the positive rate in patients with CI and ICH was nearly twice that in patients with CI or ICH alone (OR = 1.94). In future studies, the authors plan to increase the sample size of patients with CI complicated with ICH, in order to better evaluate the anti-BP180 antibody positive rate in patients with different stroke subtypes.

A limitation of this study was the absence of quantitative criteria for stroke severity. Hence, the relationship between stroke severity and anti-BP180 antibody levels could not be evaluated. Further research is needed to test whether antibody levels are also related to the severity of clinical manifestations of stroke.

In summary, this study found higher values and a positive rate of anti-BP180 antibody in stroke patients compared to the controls, indicating that stroke patients may have a greater risk of developing BP. Further follow-up is needed to determine the outcome of the anti-BP180 antibody-positive patients.

## Financial support

This work was supported by the Academic promotion program of Shandong First Medical University (2019LJ002, 2019RC007, 2020RC001), the Youth Technology Innovation Support Project of Shandong Colleges and Universities (2019KJL003) and the Innovation Project of Shandong Academy of Medical Sciences.

## Authors' contributions

Jing Wang: Formal analysis; investigation; writing – original draft; approval of final manuscript.

Hong Liu: Project administration; supervision; writing – review & editin; approval of final manuscript.

Zhenzhen Wang: Data curation; methodology; software; approval of the final manuscript.

Qing Pan: Validation; visualization; approval of the final manuscript.

Furen Zhang: Conceptualization; funding acquisition; resources; approval of the final manuscript.

## Conflicts of interest

None declared.
